# Ultrasound-Assist Extrusion Methods for the Fabrication of Polymer Nanocomposites Based on Polypropylene/Multi-Wall Carbon Nanotubes

**DOI:** 10.3390/ma8115431

**Published:** 2015-11-23

**Authors:** Carlos A. Ávila-Orta, Zoe V. Quiñones-Jurado, Miguel A. Waldo-Mendoza, Erika A. Rivera-Paz, Víctor J. Cruz-Delgado, José M. Mata-Padilla, Pablo González-Morones, Ronald F. Ziolo

**Affiliations:** 1 Centro de Investigación en Química Aplicada, Blvd. Ingeniero Enrique Reyna Hermosillo. 140, Col. San José de los Cerritos, Saltillo, Coahuila CP 25294, Mexico; victor.cruz@ciqa.edu.mx (V.J.C.-D.); jose.mata@ciqa.edu.mx (J.M.M.-P.); pablo.gonzalez@ciqa.edu.mx (P.G.-M.); ronald.ziolo@ciqa.edu.mx (R.F.Z.); 2 Innovación y Desarrollo en Materiales Avanzados A. C., Grupo POLYnnova, Carr. San Luis Potosí-Guadalajara 1510, Nivel 3, Local 12, Lomas del Tecnológico, San Luis Potosí, SLP, CP 78211, Mexico; zoe.vineth@polynnova.mx (Z.V.Q.-J.); miguel.waldo@polynnova.mx (M.A.W.-M.); erika.rivera@polynnova.mx (E.A.R.-P.)

**Keywords:** polypropylene, carbon nanotubes, melt flow index, extrusion, ultrasound, surface resistivity, electric charge

## Abstract

Isotactic polypropylenes (iPP) with different melt flow indexes (MFI) were used to fabricate nanocomposites (NCs) with 10 wt % loadings of multi-wall carbon nanotubes (MWCNTs) using ultrasound-assisted extrusion methods to determine their effect on the morphology, melt flow, and electrical properties of the NCs. Three different types of iPPs were used with MFIs of 2.5, 34 and 1200 g/10 min. Four different NC fabrication methods based on melt extrusion were used. In the first method melt extrusion fabrication without ultrasound assistance was used. In the second and third methods, an ultrasound probe attached to a hot chamber located at the exit of the die was used to subject the sample to fixed frequency and variable frequency, respectively. The fourth method is similar to the first method, with the difference being that the carbon nanotubes were treated in a fluidized air-bed with an ultrasound probe before being used in the fabrication of the NCs with no ultrasound assistance during extrusion. The samples were characterized by MFI, Optical microscopy (OM), Scanning electron microscopy (SEM), Transmission electron microscopy (TEM), electrical surface resistivity, and electric charge. MFI decreases in all cases with addition of MWCNTs with the largest decrease observed for samples with the highest MFI. The surface resistivity, which ranged from 10^13^ to 10^5^ Ω/sq, and electric charge, were observed to depend on the ultrasound-assisted fabrication method as well as on the melt flow index of the iPP. A relationship between agglomerate size and area ratio with electric charge was found. Several trends in the overall data were identified and are discussed in terms of MFI and the different fabrication methods.

## 1. Introduction

Carbon nanotube/polymer nanocomposites [1,2,3,4,5,6] have been studied for over a decade due to their outstanding properties, which result from the combination of a soft phase (polymer matrix) and a functional phase (dispersed nanomaterials). Among other properties and attributes, carbon nanoparticles can provide mechanical strength [7,8] to polymers, act as gas barriers in polymers [9,10], and effect electron transport in polymers [11]. Nevertheless, obtaining outstanding properties requires an effective dispersion of the carbon nanotubes (CNTs) in the polymer matrix, which is easily hindered by particle-particle attraction to form agglomerates and by the lack of affinity between the nanoparticles and the matrix.

To address and solve this problem, a number of fabrication methods, based on *in situ* polymerization [12,13], melt blending [14,15], and solution mixing [16,17] have been developed in order to have an adequate dispersion that optimizes the targeted properties. On the other hand, polymers, as well as CNTs, are often subjected to treatments prior to nanocomposite fabrication in order to reduce the free energy difference between the components. In this sense, the application of high-frequency sound waves during pretreatment, as well as during fabrication, could provide an effective energy source to improve the dispersion of the functional CNTs in the polymer matrix.

A few examples of this approach can be found in the literature. For example, prior to fabrication using a solution mixing method, nanoparticles in solution are often pretreated ultrasonically [18]. On the other hand, nanoparticles can be de-agglomerated, say in a beaker, using an ultrasound probe in an open air system that creates a fluidized air bed [19,20]. Thereafter, the treated nanoparticles can be used in melt-blending nanocomposite fabrication.

Another example is the direct application of ultrasound waves during melt blending in an extrusion process [21,22,23]. This is typically done at a single or fixed acoustic frequency. However, other fabrication methods use a number of different, sweep, or variable frequencies instead of a single frequency, in an expansion chamber, which seems to improve the dispersion even more [24,25]. As we noted earlier, this behavior might be related to the fact that there is a resonant frequency or frequencies that might be associated with a given length of polymer chain (vibrational motion of chains mechanism) rather than to cavitation effects [26]. Bearing in mind that the polymer chain lengths have a wide distribution, we note that a number of different frequencies is necessary to change the chain conformation from random to elongate. Once the given acoustic frequency is no longer applied, the chain conformation relaxes to the random conformation. This process or mechanism might improve chain mobility and polymer access by the CNTs and, therefore, lead to more efficient dispersion of the nanoparticles.

Up to this point, it is has not been possible to compare each of the ultrasound-assist methods since they have been used in different polymer systems or under different conditions. Given the paucity of data and difficulty in comparing different ultrasound extrusion fabrication studies, we focused on a model system to determine the effects of ultrasound-assist methodology on the properties of nanocomposites formed by the extrusion process. For this purpose, we choose a system based on isotactic polypropylene (iPP) with three different melt flow index as the matrix and on multi-walled carbon nanotubes (MWCNTs) as the functional additives. Such polymer/CNT systems have been widely studied during the last decade due to their industrial application and scientific interest. In particular, MWCNTs are excellent electric conductors, which can improve the electrical properties of non-conductive iPP, and polymer chain mobility can be monitored and controlled by using iPP with different melt flow index.

## 2. Results and Discussion

### 2.1. Melt Flow Index

The melt flow index (MFI) for iPP homopolymers and iPP/MWCNT nanocomposites fabricated with different methods are shown in Table 1. The four fabrication methods are designated as W-U for fabrication without ultrasound, F-U for fixed-frequency ultrasound fabrication, V-U for variable-frequency ultrasound fabrication and PT for pretreated carbon nanotubes, where the carbon nanotubes were treated in a fluidized air-bed with an ultrasound probe before being used in the fabrication of the nanocomposites (NCs), with no ultrasound assistance during the extrusion. The usual designation for multi-wall carbon nanotubes (MWCNTs) was shortened to MNT and used hereafter.

The results are explained bearing in mind the power law relationship between MFI and molecular weight, where a steep decay in molecular weight is observed at low MFI, while minor changes in molecular weight are observed at intermediate and high MFI [27,28,29]. MFI for the as-received iPP homopolymers was evaluated prior to nanocomposite fabrication. The values obtained in this study were the average value of measurements taken on five different portions of each suppliers’ iPP and agree well with the suppliers’ value with only minor deviations. In the case of iPP_MFI = 2.5_, a slight increase in MFI is obtained after processing in normal conditions, *i.e.*, fabrication without ultrasound-assist (W-U), probably due to chain scission. Nonetheless, the use of ultrasound waves seems to decrease the MFI. This can be due to the fact that ultrasound waves can either break or bond some of the polymer chains. In the latter case, an increase in the chain length would indeed diminish homopolymer fluidity. For samples with a higher MFI, iPP_MFI = 34_, the extrusion processing effect and the use of ultrasound waves do not seem to affect, in a marked manner, the fluidity of the sample. Finally, melt extrusion of iPP_MFI = 1200_ seems to reduce its fluidity probably due to the bonding of short polymer chains. Furthermore, in this particular case, ultrasound application seems to reduce chain length thus increasing MFI.

Regardless of the fabrication method, the MFI for iPP/MNT nanocomposites is much lower compared to the pure iPP homopolymers. In the case of nanocomposites fabricated with homopolymers iPP_MFI = 2.5_ and iPP_MFI = 34_ the MFI values obtained are close to half of the values obtained for the pure homopolymers, while in the case of iPP_MFI = 1200_ the values for the nanocomposites are up to ten times smaller. In a first approach, it can be considered that MNTs act as mobility barriers hindering the transport of polymer chains, thus increasing viscosity [28]. Thus, MFI tends to decrease with the presence of a large amount (10 wt %) of MNTs. Nevertheless, the complexity of processing imposes barriers for a full understanding of the system, regarding the effect of the ultrasound-assist fabrication method.

**Table 1:** Melt Flow Index (g/10 min) for isotactic polypropylenes (iPP) homopolymers, and iPP/multi-wall carbon nanotubes (MNT) nanocomposites fabricated by different methods.

Sample	Fabrication Method				
	As Received	W-U	F-U	V-U	PT
iPP_MFI = 2.5_	2.70	3.00	2.91	2.85	3.00
iPP_MFI = 34_	34.34	35.60	37.57	35.73	35.60
iPP_MFI = 1200_	1208.87	1013.27	1250.85	1281.86	1013.27
iPP_MFI = 2.5_/MNT	-	1.49	1.59	1.55	1.42
iPP_MFI = 34_/MNT	-	17.53	18.63	18.50	18.40
iPP_MFI = 1200_/MNT	-	179.40	159.12	171.61	160.01

### 2.2. Morphology

**Figure 1:**
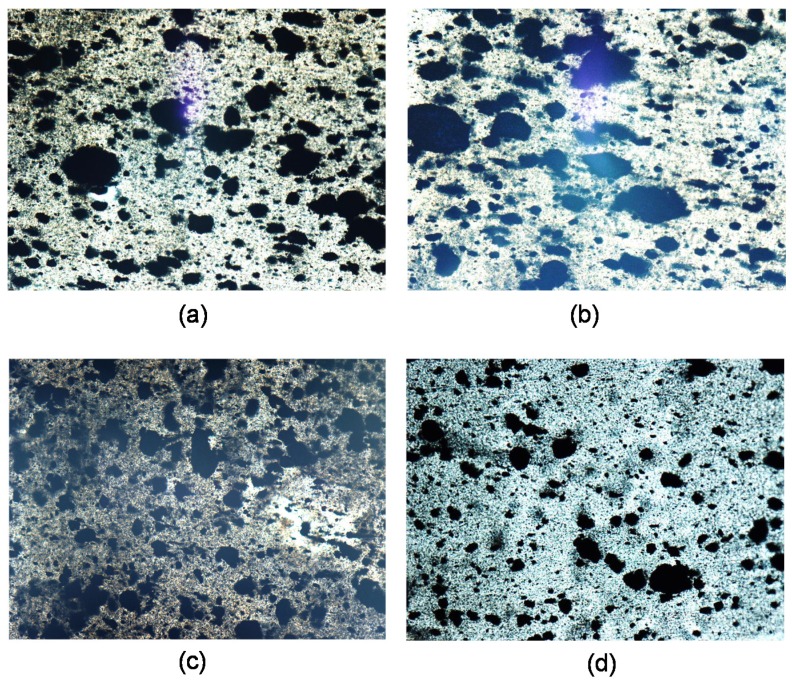
Optical micrographs for nanocomposites for isotactic polypropylenes/multi-wall carbon nanotubes (iPP/MNT) (10 wt %) fabricated by melt extrusion with and without ultrasound-assist fabrication for iPP_MFI = 2.5_. (**a**) W-U; (**b**) F-U; (**c**) PT; (**d**) V-U.

The morphology of iPP/MNT polymer nanocomposites was analyzed in terms of the MNT agglomerate size, the area ratio of the MNT agglomerates, and dispersion of the MNTs in the iPP matrix. Although MNTs have a diameter in the scale of nanometers, their length is typically on the order of 1–20 μm and they tend to agglomerate due to van der Waals forces. Thus, it is important to characterize the size of agglomerates to determine the effect of ultrasound-assist fabrication. Optical microscopy micrographs are shown in Figure 1 for samples of iPP_MFI = 2.5_ prepared using different fabrication methods. The black spots correspond to MNT agglomerates, while the white/greyish colors correspond to the polymer.

The figure shows that the MNT agglomerates are markedly larger for the methods W-U and F-U than for methods PT and V-U; nonetheless, the direct observation of large agglomerates can lead to erroneous conclusions. Thus, in order to have a numerical comparison, micrographs for each method were analyzed, and the results are shown in Table 2. The agglomerate size of the MNTs in the polymer nanocomposites was close to 10 μm with a few deviations. Menzer [30] obtained similar values for as-received and ball-milled shortened carbon nanotubes. The lowest agglomerate size, 8.56 μm, was determined for the sample iPP_MFI = 2.5_ prepared using the V-U method, while the highest value of 12.95 μm was obtained for iPP_MFI = 34_ using the PT method. In general, for the different homopolymers, agglomerate size is practically the same when no ultrasound is used, independent of the MFI. However, in the case of samples prepared with ultrasound-assist fabrication, either with fixed or variable frequency, the lower values were obtained for samples with the lowest MFI. Thus, the shear force of long polymer chains generated during melt extrusion seem to help agglomerate breaking, whereas the shear force of short polymer chains does not. Additionally, it can be noticed that a combination of high MFI (iPP_MFI = 1200_) using the V-U method results in a large agglomerate size. On the other hand, the MNT area ratio does not show a clear tendency (Table 3). Nonetheless, we note that the samples prepared using V-U show the lowest values, while iPP_MFI = 2.5_ prepared using PT show the highest value. It is also worth noting that increasing the fluidity of the sample the MNT area ratio does not change markedly in the cases of samples prepared with W-U and F-U. However, in the case of samples prepared using V-U, it can be noticed that increasing the MFI, the area ratio increases, while in the case of PT the values decrease. This behavior suggests that both, the combination of low fluidity homopolymer plus variable ultrasound in the former, and the use of high fluidity homopolymer with de-agglomerated MNT (without ultrasound), are effective ways to reduce the MNT area ratio.

**Figure 2:**
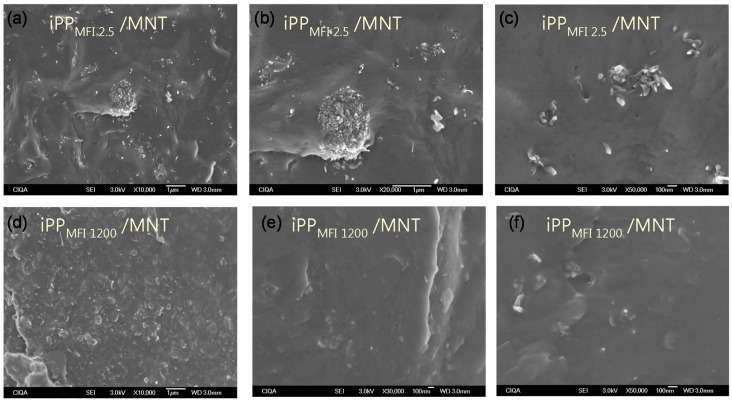
Scanning electron microscopy (SEM) micrographs of the surface fracture for nanocomposites of iPP/MNT (10 wt %) fabricated by melt extrusion without ultrasound-assist fabrication. (**a**) iPP_MFI2.5_/MNT at 10K magnification; (**b**) iPP_MFI2.5_/MNT at 20K magnification; (**c**) iPP_MFI2.5_/MNT at 50K magnification; (**d**) iPP_MFI1200_/MNT at 10K magnification; (**e**) iPP_MFI1200_/MNT at 30K magnification; (**f**) iPP_MFI1200_/MNT at 50K magnification.

On the other hand, the morphology of polymer nanocomposites was observed on the surface of fractured samples prepared by W-U (Figure 2). Agglomerates of 1 μm as well as individual MNTs are seen for iPP_MFI = 2.5_/MNT, while for iPP_MFI = 1200_/MNT only individual MNTs are observed. This behavior suggests that extrusion of high viscosity samples is not enough to break apart agglomerates, while the use of a low viscosity polymer helps to disentangle MNT bundles [28].

**Table 2:** Agglomerate size (μm) for iPP/MNT nanocomposites fabricated by different methods.

iPP_MFI_/MNT MFI (g/10 min)	Fabrication Method			
	W-U	F-U	V-U	PT
2.5	10.14 (±2.12)	9.19 (±2.51)	8.56 (±5.40)	9.47 (±2.73)
34	9.60 (±1.02)	9.70 (±2.15)	10.63 (±1.21)	12.95 (±0.80)
1200	10.09 (±0.76)	10.35 (±1.46)	12.02 (±2.69)	10.11 (±2.98)

**Table 3:** Area ratio for iPP/MNT nanocomposites fabricated by different methods.

iPP_MFI_/MNT MFI (g/10 min)	Fabrication Method			
	W-U	F-U	V-U	PT
2.5	37.96 (±9.89)	33.97 (±10.01)	11.18 (±6.78)	57.34 (±7.95)
34	41.16 (±7.69)	35.22 (±8.30)	17.45 (±2.07)	31.46 (±13.06)
1200	33.79 (±5.42)	35.48 (±6.27)	22.65 (±4.24)	24.29 (±2.41)

### 2.3. Electrical Behavior

Electrical surface resistivity (SR) and electrostatic charge of the iPP homopolymers and polymer nanocomposites were measured for the three different MFIs and fabrication methods and evaluated within the limits of experimental measurement procedures.

#### 2.3.1. Surface Resistivity

Electrical surface resistivity (SR) values of the iPP homopolymers and polymer nanocomposites are presented in Table 4. SR values for the iPP polymers appear independent of the MFI and show the polymers to be insulators at ≥10^13^ Ω/sq as expected. The SR values for the iPP_MFI = 2.5_/MNT samples, however, are at least an order of magnitude less, at 10^12^ Ω/sq, regardless of the fabrication method. Although the samples are insulators, they appear to be able to transport more charge than the iPP homopolymers.

**Table 4:** Surface resistivity (Ω/sq) for iPP homopolymers, and iPP/MNT nanocomposites fabricated by different methods.

Sample	Fabrication Method			
	W-U	F-U	V-U	PT
iPP_MFI = 2.5_	≥10^13^	-	-	-
iPP_MFI = 34_	≥10^13^	-	-	-
iPP_MFI = 1200_	≥10^13^	-	-	-
iPP_MFI = 2.5_/MNT	10^12^/(I)	10^12^/(I)	10^12^/(I)	10^12^/(I)
iPP_MFI = 34_/MNT	10^12^/(I)	10^9^/(SD)	10^6^/(SD)	10^5^/(C)
iPP_MFI = 1200_/MNT	10^5^/(C)	10^6^/(SD)	10^8^/(SD)	10^5^/(C)

I = Insulator material; SD = static dissipative material; C = conductive material.

A drastic change in SR is observed for the samples with higher MFI, where a marked decrease in resistivity is observed. For example, for samples iPP_MFI = 34_/MNT, a decrease of up to seven orders of magnitude is observed when the sample is prepared by the PT fabrication method (10^5^ Ω/sq) to give a slightly more conductive material. Application of ultrasound waves in the melt also decreases the SR, which is more pronounced when a variable frequency is applied (10^6^ Ω/sq) than when a fixed frequency is used (10^9^ Ω/sq). In this case, however, the materials produced are static-dissipative. On the contrary, the sample prepared without ultrasound assist shows an SR of 10^12^ Ω/sq compared with the sample with lower MFI and is also an insulator.

For samples of iPP_MFI = 1200_/MNT, a remarkable reduction in electrical resistivity is observed (10^5^ Ω/sq) for the sample prepared without ultrasound-assist as compared to samples with lower MFI using the same fabrication method. The same result was obtained for samples prepared by the PT method, which already showed this resistivity level for samples with MFI of 34 g/10 min. In the case of ultrasound-assisted methods, slightly higher values were obtained, *i.e.*, for static dissipative materials.

A wide range of electrical surface resistivity values is found in the literature for iPP/MNT systems prepared using melt blending. Since reported electrical resistivity or conductivity values can refer to volume or surface resistivity, we converted the reported data to electrical surface resistivity for comparison purposes. For example, Steinmann [31] reported a value of 10^2^ Ω/sq for MNT loadings of 7.5 wt % using an iPP with MFI of 25 g/10 min, while Wang [32] used an iPP with an MFI of 1.2 obtaining a value of 10^9^ Ω/sq. 

Although it might be argued that MFI plays an important solitary role in determining SR, it is known that a number of factors contribute to the final surface resistivity of the material, such as (1) the intrinsic properties of the iPP such as the molecular weight, which is intimately linked to MFI; (2) the MNT type, outer diameter, length, purity, agglomerate size, *etc.*; and (3) the fabrication parameters including type of machine, temperatures, shear rates, *etc.* In this regard, different authors [33,34,35] reported values of 10^5^ Ω/sq for MNT concentrations *ca.* 5–6 wt % and each of them used different MFI, types of nanotubes and processing parameters. Finally, Pan [36] used a 10 wt % concentration of MNT in iPP with an MFI of 35 g/10 min that showed an electrical resistivity of 10^6^ Ω/sq. The materials used in Pan’s study are at least comparable to the ones used here, as are the reported SRs. Thus, the experimentally-observed SR values in this study compare favorably with SR values reported by other authors.

From the SR results obtained in this study, we note some trends that merge regarding the fabrication method and MFI of iPP NCs. First of all, insulator-like materials are obtained when a low MFI of 2.5 g/10 min is used, regardless of the fabrication method. The fabrication method, however, plays a key role in decreasing the electrical resistivity when higher MFI polypropylenes (MFI = 34 and 1200 g/10 min) are used, where static dissipative and mildly-conductive materials are obtained. For a discussion of the transition from insulating to conducting materials for samples prepared using solution blending, see [18].

Static-dissipative materials were obtained for samples fabricated with ultrasound-assist methods in the melt, while insulator and conductive materials were obtained when samples were fabricated without the presence of ultrasound waves in the melt. In the case of samples fabricated without ultrasound-assist, an insulator-like material is obtained for an MFI of 34 g/10 min, while a slightly conductive material is obtained when the MFI is 1200 g/10 min. Finally, mildly-conducting materials were obtained when the MNTs were de-agglomerated in the fluidized air bed for both MFIs.

#### 2.3.2. Electrical Charge

It is well know that vinyl polymers, such as isotactic polypropylene, can be easily charged via friction rubbing, for example, with a wool fabric. Following this simple methodology, we used 56 mm by 0.1 mm square plaques of iPP homopolymers and iPP/MNT NCs and rubbed them following the empirical procedure described in the experimental section to test if they could acquire and sustain an electrical charge. Charge results in kV are shown in Table 5. It is seen that the iPP homopolymers are charged negatively from their initial charged state. These values represent an average of three individual measurements on each sample and appear to trend more negatively for higher MFI.

**Table 5:** Electric charge (kV) for iPP homopolymers, and iPP/MNT nanocomposites fabricated by different methods.

Sample	Fabrication Method			
	W-U	F-U	V-U	PT
iPP_MFI = 2.5_	0.00/−0.40	-	-	-
iPP_MFI = 34_	0.33/−0.48	-	-	-
iPP_MFI = 1200_	−0.02/−1.22	-	-	-
iPP_MFI = 2.5_/MNT	0.00/−0.25	0.04/−0.10	−0.01/−1.04	0.00/−0.03
iPP_MFI = 34_/MNT	0.00/0.00	0.00/0.00	0.00/0.00	0.00/0.00
iPP_MFI = 1200_/MNT	0.00/0.00	0.00/0.00	0.00/0.00	0.00/0.00

The slash (/) indicates charge before and after friction rubbing.

On the other hand, samples fabricated using iPP_MFI = 2.5_/MNT appear to charge negatively after rubbing, with the final charge depending on the fabrication method. The initial charge ranges from 0 to +40 V, while the final charge ranges from −30 to −1040 V. The lowest final charge was obtained for samples prepared using the PT method, while the V-U method rendered samples with the highest charge. The values obtained for samples prepared using the W-U and F-U methods were −250 and −100 V, respectively.

Interestingly, the electric charge measured for all of the iPP_MFI = 34_/MNT and iPP_MFI = 1200_/MNT NCs was zero within the time frame of the measurement, irrespective of the fabrication method. These observations can be explained with the aid of the so-called MNT area ratio, which is the ratio of the area of the MNT agglomerates to that of the sample as determined by optical microscopy, and the surface resistivity results.

**Figure 3:**
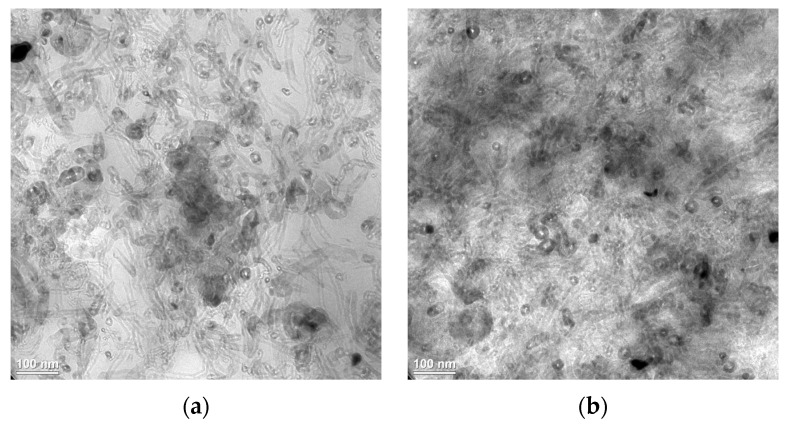
TEM micrographs of a section for nanocomposites of iPP_MFI = 2.5_/MNT (10 wt % fabricated by melt extrusion (**a**) without, and (**b**) with, V-U ultrasound-assist fabrication.

As expected, iPP can be easily charged since they are insulating materials. However, the electric charge of samples prepared using iPP_MFI = 2.5_/MNT appears to depend on the fabrication method. The lowest value was obtained using de-agglomerated MNT in the gas phase (PT) while the highest value was obtained for the method that used variable frequencies in the molten state (V-U). Intermediate values are found for the two remaining methods. This behavior can be associated with the MNT area ratio, where the highest value was obtained for PT samples, and the lowest for the V-U samples, while intermediate values were found for the other two methods. Thus, it appears that a correlation emerges between MNT area ratio and the electric charge at low MFI, *i.e.*, a large area ratio means that the surface of the sample is covered with a greater amount of carbon nanotubes that are not easily charged, while a small value suggests that large portions of iPP can be charged. It is not surprising to note that even though the SR values for these samples are all 10^12^ Ω/sq, indicating their insulator nature, the materials behave differently in terms of electrical charge, which relates to triboelectric effects. To support this idea, TEM images were collected for the sample iPP_MFI = 2.5_/MNT prepared using V-U and the one prepared using W-U (see Figure 3). In terms of the extrusion process, we believe that both samples have no difference right before the influence of the ultrasound waves. Once the sample enters into the influence of ultrasound, a change in behavior is expected, as seen in Section 2.2. Many images were collected and showed a trend that untreated samples (W-U) are more loosely packed than the treated ones (V-U). We speculate that during the ultrasound irradiation, individual MNT are displaced from the agglomerates by erosion and end up in the polymer phase where there is a minimal concentration of CNTs, but at the same time, increasing the density of packaging. A larger distance separates these densely packaged agglomerates (see area ratio in Table 3), then showing a charge behavior. However, the agglomerates that were untreated (W-U) were not prone to show this effect and maintained a large agglomerate size with minor distance between agglomerates (see area ratio in Table 3).

Finally, we note that regardless of the MFI used and the fabrication method, samples prepared with iPP with MFI of 34 and 1200 g/10 min showed no initial and no final electrical charge, even for the sample iPP_MFI = 34_/MNT prepared using W-U that show insulating behavior, and for samples with static-dissipative values. The results obtained in this study suggest that both electrical resistivity and electrical charge are necessary to determine the electrical behavior for hydrophobic materials such as iPP/MNT NCs.

## 3. Experimental Section

Materials: Three iPP homopolymers with different MFI were used. The homopolymers were provided by Exxon (Houston, TX, USA), grade PP4712 E1 (MFI = 2.5 g/10 min), Indelpro grade PL835N (MFI = 34 g/10 min) and, finally, Indelpro grade Profax PL 505 (MFI = 1200 g/10 min). These three materials were designated as iPP_MFI = 2.5_, iPP_MFI = 34_ and iPP_MFI = 1200_, according to their manufacturer reported MFI. Multiple-wall carbon nanotubes grade IGCNTs were purchased from Cheaptubes, Cambridgeport, VT, USA, with an outer diameter of 20–40 nm, length 10–30 μm, and 90% purity.

Methods: iPP/MNT nanocomposites with 10 wt % of MNT were fabricated using a laboratory twin-screw extruder model 24MC (Thermo Scientific, Stone, UK) with an L/D = 40:1, a speed of 100 rpm, and a plain temperature profile of 220 °C along the barrel. iPP pellets and MNT powder were fed at the main feeder of the extruder at a feed rate of 2 kg/h and 200 g/h, respectively. A specially-designed expansion chamber for ultrasonic treatment was adapted at the die zone and kept at 220 °C, which allowed ultrasound application in the melt. Details of this device were reported elsewhere [23]. The 750 W ultrasound generator was made in-house and can perform a sweep function to scan a frequency range with discrete increments [24].

Four different fabrication methods were used to fabricate the polymer nanocomposites. In the first method, the processing of the materials was carried out with no ultrasound-assist and materials were used as received. This method was designated W-U for “without ultrasound”. In the second and third methods, the materials were used as received, however the ultrasound generator was switched on in both cases, and a commercial transducer from Advanced Sonic Processing Systems with a main frequency of 20 ° 0.2 kHz was used. A fixed frequency of 20 ± 0.1 kHz was used in the second method, while a frequency scan of 20–50 kHz (with 0.1 scan step and 250 cycles per scan) was used in the third method. In both cases the generator output was 500 W. These methods were designated as F-U for fixed frequency ultrasound-assist fabrication and V-U for variable-frequency ultrasound-assist fabrication. The fourth method was similar to the first method in the sense that the materials were not subjected to ultrasound during melt extrusion. However, the MNTs were treated in a fluidized air-bed with an ultrasound probe before being used in the fabrication of the NCs. This method promotes the size reduction of the MNT agglomerates and was designated as PT for pretreatment.

Molded plaques 15 cm × 15 cm × 0.1 mm were obtained in a mold using a hydraulic compression press model Q230H-X4A (PHI, Industry, CA, USA) at 200 °C. Molded plaques were cut into 56 mm squares and were conditioned at 23 °C and 50% RH 50% during 48 h to homogenize the samples prior to evaluation at ambient conditions.

### 3.1. Characterization Techniques

*Melt Flow Index (MFI).* Melt flow of materials was evaluated using an extrusion plastometer in accordance with the ASTM D 1238 standard method [37]. For this purpose a Kayeness Melt Indexer was used at 230 °C, with a weight of 2.16 kg. Five measurements were made and the average value is reported.

*Optical Microscopy (OM).* This technique was used to determine the size (mean diameter, *D*_m_) of agglomerate in iPP/MNT nanocomposites made using the four different fabrication methods. Image Pro Plus software from Media Cybernetics (Rockville, MD, USA), was used to analyze the images. OM observations were performed in an optical microscope, Olympus BX53 (Tokyo, Japan).

*Scanning Electron Microscopy (SEM).* SEM observations were used to determine the nanostructure within the matrix of the polymer nanocomposites. Pieces of compression molded plaques were cryo-fractured and coated with gold. The SEM observations were realized directly on the external surface and cryo-fractured surface of the iPP/MNT nanocomposites fabricated without ultrasound (W-U). SEM micrographs were obtained using a field emission scanning electron microscope, JSM-74101F- JEOL VR (JEOL, Tokio, Japan) with a secondary electron detector (SEI) using a voltage of 4.0 kV.

*Electrical Surface Resistivity.* This property was measured on homopolymers and polymer nanocomposites using the 56 mm square molded plaques described above and a Surface Resistance Meter from Antistat (Ipswich, UK) model ANT093-0050. The apparatus was placed on the surface of the molded plaques for 30 s; each sample was measured three time and the values averaged.

*Electrostatic Charge.* Electrostatic charge was evaluated with a portable electrostatic field meter Simco-Ion model FMX-004 (Simco Ion, Industrial Group, Hatfiel, PA, USA), in the low range mode (0–3.00 kV). The same samples employed for electrical resistance were used in this test and three independent measurements were carried out on each sample and averaged. The samples were left to equilibrate for 2 h in a small open chamber protected from dust and draft at ambient conditions (23 °C, 40% RH and an O_2_ partial pressure of ~135 mm Hg in Saltillo). Each sample was clamped to a grounded steel ring and friction rubbed edge to edge once with a clean wool fabric cloth applying a steady pressure.

*Transmission Electron Microscopy (TEM)*. TEM was used to analyze the inner morphology of MNT agglomerates in the PP/MNT nanocomposites. Thin sections (~90 nm) of compression molded plaques of PP_MFI=2.5_/MNT, prepared without ultrasound (W-U) and variable frequency ultrasound (V-U), were cryo-microtomed. TEM micrographs were obtained using a TEM Titan 89-300 (FEI, Hillsboro, OR, USA).

### 3.2. Complementary Characterization Techniques. 

Polypropylene homopolymers with different melt flow indexes (2.5, 34 and 1200 g/10 min) and iPP/MNT nanocomposites prepared by different methodologies were analyzed by complementary techniques as follows. (Results shown in the Supplementary Materials).

*Thermogravimetric Analysis (TGA)*. TGA was used to analyze the thermal behavior. Analyses were performed using a Q500 analyzer (TA Instruments, Newcastle, DE, USA) with a heating rate of 10 °C/min and gas flow rate of 50 mL/min. Sample runs were carried out from 40 to 600 °C under an N_2_ atmosphere and under an O_2_ atmosphere above 600 °C. The results are shown in Figure S1 and Tables S1, S2 and S3 of Supplementary Materials.

*Differential Scanning Calorimetry (DSC)*. A DSC model Discovery from TA Instruments was used to analyze the melting and crystallization behavior. The analyses were performed at a heating and cooling rate of 10 °C/min in the range of 25–200 °C. Pieces of each material were weighed (8 ± 1 mg) and sealed within aluminum sample pans before experiments. The equipment was calibrated with an indium standard in an N_2_ atmosphere. The results are shown in Figure S2 and Tables S4, S5 and S6 of Supplementary Materials.

*Dynamic Mechanical Analysis (DMA)*. Dynamic mechanical properties (storage modulus (E’), loss modulus (E’’), and damping, tan δ, which is the ratio of E’’/E’), were determined using a dynamic mechanical analyzer model Q800 (TA Instruments). Rectangular sections of compression-molded parts (3 cm × 1.2 cm × 0.1 cm) were used to perform the analysis. Each analysis was carried out at a heating rate of 3 °C/min from −50 to 120 °C, an oscillation amplitude of 0.3 to 0.5 mm, and a frequency of 1 Hz. The results are shown in Figure S3 and Tables S7, S8 and S9 of Supplementary Materials.

## 4. Conclusions

The present study showed that methods used for ultrasound-assist extrusion fabrication influence the morphology and electrical properties of isotactic polypropylenes (iPP) with different MFI and of nanocomposites (NCs) with 10 wt % loadings of multi-wall carbon nanotubes using the same polymers. Four fabrication methods were studied and compared, two with variable and fixed frequency ultrasound and two with no ultrasound assist during extrusion. The MFI of the NCs decreased to half the value for low (2.5 g/10 min) and medium (34 g/10 min) MFI polypropylene, and an order of magnitude for high (1200 g/10 min) MFI resin, and appeared independent of the NC fabrication method. The lowest agglomerate size was found for the NC prepared with iPP_MFI = 2.5_ using variable frequency assist; at the same time, this sample showed the lowest agglomerate area ratio, consistent with the observed electrical behavior where large polymer areas exist with a minimal presence of carbon nanotubes. Surface resistivity values for these samples were in the insulator range; thus, the samples were able to sustain electric charge by friction rubbing. The value of the electric charge appeared dependent on the fabrication method. The NC prepared with iPP_MFI = 2.5_, using the variable frequency assist fabrication method, showed the highest charge, 1040 V. For NCs with higher MFI, the resistivity decreased up to seven orders of magnitude (10^5^ Ω/sq) and was dependent on the fabrication method. Surface resistivity values were ≥10^13^ Ω/sq for the pure polymers and 10^12^ Ω/sq for iPP_MFI=2.5_ NCs independent of the fabrication method. Pure polymers were easily charged negatively by friction rubbing with wool. Nanocomposites prepared with the higher MFI values showed no charging regardless of fabrication method, consistent with their more conductive nature. The observed trends in the electrical behavior are consistent with the nanocomposite morphology.

## Supplementary Materials

The following are available online at www.mdpi.com/1996-1944/8/11/5431/s1.
