# Supplementary material for: Ultrasound-Assist Extrusion Methods for the Fabrication of Polymer Nanocomposites Based on Polypropylene/Multi-Wall Carbon Nanotubes
Source: Materials (Basel). 2015 Nov 23;8(11):7900–12. doi: 10.3390/ma8115431 (PMC5458917; doi:10.3390/ma8115431)
Supplement: Supplementary file 1 [file materials-08-05431-s001.pdf]

Supplementary Materials

In order to simplify the redaction of the manuscript, the usual designation for multi-wall carbon nanotubes (MWCNTs) was shortened to MNT and used hereafter.

Additionally, four different fabrication methods were used to fabricate the polymer nanocomposites: W-U (without ultrasound), F-U (Fixed frequency ultrasound-assist fabrication), V-U for variable-frequency ultrasound-assist fabrication and PT (Pretreatment of MNT in a fluidized air-bed with an ultrasound probe).

Thermogravimetric Analysis (TGA). Thermogravimetric analysis for isotactic polypropylenes (iPP) homopolymers and iPP/MNT nanocomposites fabricated by different methods.

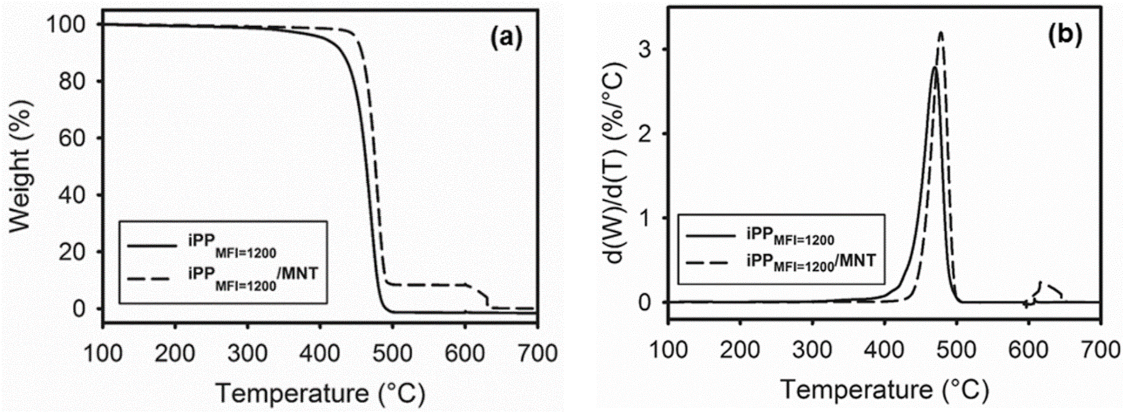

Figure S1. Thermogravimetric analysis (TGA) (**a**) and weight derivative (**b**) for isotactic polypropylenes (iPP) homopolymer fabricated by W-U and iPP/MNT nanocomposite fabricated by V-U.

Table S1. Degradation temperatures at 5% weight loss for iPP homopolymers and iPP/MNT nanocomposites fabricated by different methods.

Sample	Fabrication Method			
	W-U	F-U	V-U	PT
$iPP_{MFI=2.5}$	426.87	-	-	-
$iPP_{MFI=34}$	419.22	-	-	-
$iPP_{MFI=1200}$	405.54	-	-	-
$iPP_{MFI=2.5}$/MNT	455.76	455.48	455.19	432.87
$iPP_{MFI=34}$/MNT	452.92	452.52	452.48	439.64
$iPP_{MFI=1200}$/MNT	449.98	449.73	449.74	437.96

Table S2. Degradation temperatures at 50% weight loss for iPP homopolymers and iPP/MNT nanocomposites fabricated by different methods.

Sample	Fabrication Method			
	W-U	F-U	V-U	PT
$iPP_{MFI=2.5}$	465.27	-	-	-
$iPP_{MFI=34}$	464.62	-	-	-
$iPP_{MFI=1200}$	463.74	-	-	-
$iPP_{MFI=2.5}$/MNT	478.12	477.50	477.37	459.34
$iPP_{MFI=34}$/MNT	477.77	477.15	477.57	464.95
$iPP_{MFI=1200}$/MNT	475.99	476.23	476.25	463.35

Table S3. Degradation temperatures at peak weight loss for iPP homopolymers and iPP/MNT nanocomposites fabricated by different methods.

Sample	Fabrication Method			
	W-U	F-U	V-U	PT
$iPP_{MFI = 2.5}$	468.94	-	-	-
$iPP_{MFI = 34}$	468.36	-	-	-
$iPP_{MFI = 1200}$	469.22	-	-	-
$iPP_{MFI = 2.5}$/MNT	480.03	479.49	479.12	460.42
$iPP_{MFI = 34}$/MNT	479.59	479.06	479.23	466.85
$iPP_{MFI = 1200}$/MNT	478.15	478.39	478.16	463.93

Differential Scanning Calorimetry (DSC). Differential scanning calorimetry for iPP homopolymers and iPP/MNT nanocomposites fabricated by different methods.

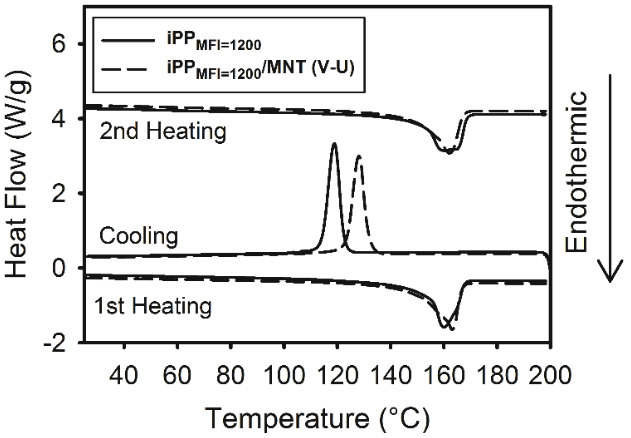

Figure S2. Differential scanning calorimetry (DSC) traces for iPP homopolymer fabricated by W-U and iPP/MNT nanocomposite fabricated by V-U.

Table S4. Melting temperature (°C)|enthalpy (J/g) during first heating for iPP homopolymers and iPP/MNT nanocomposites fabricated by different methods.

Sample	Fabrication Method			
	W-U	F-U	V-U	PT
$iPP_{MFI = 2.5}$	157.7 | 99.3	-	-	-
$iPP_{MFI = 34}$	166.3 | 95.9	-	-	-
$iPP_{MFI = 1200}$	164.4 | 102.5	-	-	-
$iPP_{MFI = 2.5}$/MNT	158.6 | 89.8	159.3 | 100.3	159.4 | 92.8	158.4 | 90.8
$iPP_{MFI = 34}$/MNT	164.5 | 95.1	165.0 | 92.8	164.5 | 99.5	163.4 | 94.2
$iPP_{MFI = 1200}$/MNT	162.5 | 101.3	162.3 | 104.8	161.9 | 100.0	161.4 | 100.0

Table S5. Crystallization temperature (°C)|enthalpy (J/g) during cooling for iPP homopolymers and iPP/MNT nanocomposites fabricated by different methods.

Sample	Fabrication Method			
	W-U	F-U	V-U	PT
$iPP_{MFI = 2.5}$	115.8 | 99.8	-	-	-
$iPP_{MFI = 34}$	117.2 | 102.5	-	-	-
$iPP_{MFI = 1200}$	118.9 | 103.5	-	-	-
$iPP_{MFI = 2.5}$/MNT	123.9 | 95.2	123.2 | 98.2	123.0 | 91.6	123.7 | 95.9
$iPP_{MFI = 34}$/MNT	126.6 | 98.3	126.3 | 94.6	126.6 | 99.0	126.6 | 98.5
$iPP_{MFI = 1200}$/MNT	127.8 | 102.6	128.6 | 104.7	128.1 | 100.6	127.7 | 103.3

Table S6. Melting temperature (°C) | enthalpy (J/g) during second heating for iPP homopolymers, and iPP/MNT nanocomposites fabricated by different methods.

Sample	Fabrication Method			
	W-U	F-U	V-U	PT
$iPP_{MFI = 2.5}$	158.2 | 96.6	-	-	-
$iPP_{MFI = 34}$	162.8 | 101.8	-	-	-
$iPP_{MFI = 1200}$	165.0 | 107.9	-	-	-
$iPP_{MFI = 2.5}$/MNT	159.7 | 94.64	159.8 | 100.9	159.8 | 93.5	159.9 | 97.9
$iPP_{MFI = 34}$/MNT	165.5 | 99.8	165.8 | 95.6	165.3 | 100.8	164.5 | 100.7
$iPP_{MFI = 1200}$/MNT	163.0 | 102.9	162.8 | 109.2	163.1 | 103.5	163.0 | 106.2

Dynamic Mechanical Analysis (DMA). Dynamical mechanical analysis for iPP homopolymers and iPP/MNT nanocomposites fabricated by different methods.

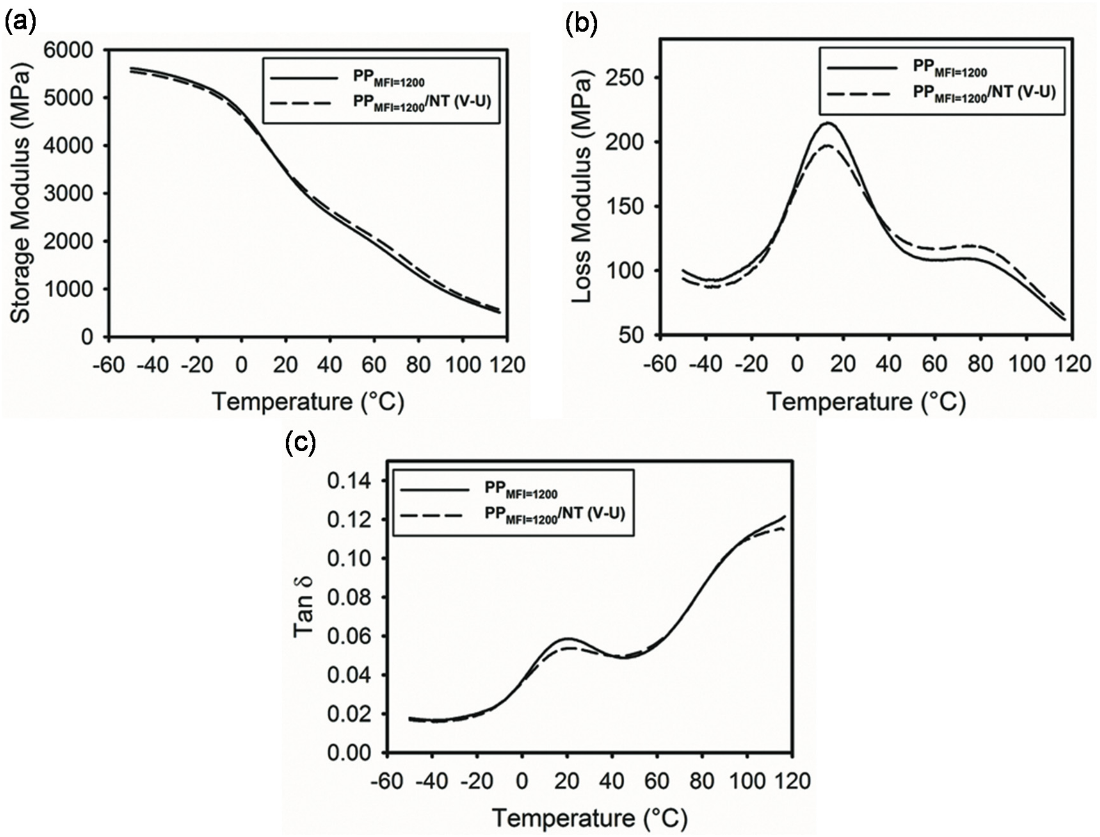

Figure S3. Dynamic mechanical analysis (DMA) results for iPP homopolymer fabricated by W-U and iPP/MNT nanocomposite fabricated by V-U. (**a**) Storage Modulus *vs.* Temperature; (**b**) Loss Modulus *vs.* Temperature; (**c**) Tan δ *vs.* Temperature.

Table S7. Storage modulus (MPa) | Loss modulus (MPa) evaluated at −50 °C for iPP homopolymers, and iPP/MNT nanocomposites fabricated by different methods.

Sample	Fabrication Method			
	W-U	F-U	V-U	PT
$iPP_{MFI = 2.5}$	5579 | 109.6	-	-	-
$iPP_{MFI = 34}$	4710 | 95.0	-	-	-
$iPP_{MFI = 1200}$	5616 | 100.0	-	-	-
$iPP_{MFI = 2.5}$/MNT	7213 | 139.0	5255 | 108.2	6290 | 114.7	5707 | 114.4
$iPP_{MFI = 34}$/MNT	6116 | 117.4	6052 | 104.7	5931 | 119.6	5750 | 104.2
$iPP_{MFI = 1200}$/MNT	6452 | 107.6	5960 | 101.6	5544 | 93.3	5735 | 93.3

Table S8. Loss modulus (MPa) evaluated at 18 °C | Loss modulus evaluated at 80 °C for iPP homopolymers and iPP/MNT nanocomposites fabricated by different methods.

Sample	Fabrication Method			
	W-U	F-U	V-U	PT
$iPP_{MFI=2.5}$	210.8 | 96.1	-	-	-
$iPP_{MFI=34}$	174.5 | 88.1	-	-	-
$iPP_{MFI=1200}$	214.9 | 107.8	-	-	-
$iPP_{MFI=2.5}$/MNT	265.1 | 134.3	190.7 | 99.7	225.2 | 114.9	205.1 | 105.1
$iPP_{MFI=34}$/MNT	212.4 | 121.2	194.9 | 114.8	201.7 | 112.7	194.5 | 109.5
$iPP_{MFI=1200}$/MNT	214.8 | 134.3	207.0 | 121.5	197.1 | 118.1	197.3 | 121.2

Table S9. Glass transition temperature (°C) obtained from tan δ *vs.* T for iPP homopolymers and iPP/MNT nanocomposites fabricated by different methods.

Sample	Fabrication Method			
	W-U	F-U	V-U	PT
$iPP_{MFI=2.5}$	19.4	-	-	-
$iPP_{MFI=34}$	22.7	-	-	-
$iPP_{MFI=1200}$	19.9	-	-	-
$iPP_{MFI=2.5}$/MNT	20.6	19.4	19.6	20.8
$iPP_{MFI=34}$/MNT	23.2	22.5	21.3	23.4
$iPP_{MFI=1200}$/MNT	18.9	18.0	22.0	20.4
